# Can Proximal Junctional Kyphosis after Surgery for Adult Spinal Deformity Be Predicted by Preoperative Dynamic Sagittal Alignment Change with 3D Gait Analysis? A Case–Control Study

**DOI:** 10.3390/jcm11195871

**Published:** 2022-10-04

**Authors:** Tomoyuki Asada, Kousei Miura, Masao Koda, Hideki Kadone, Toru Funayama, Hiroshi Takahashi, Hiroshi Noguchi, Yosuke Shibao, Kosuke Sato, Fumihiko Eto, Kentaro Mataki, Masashi Yamazaki

**Affiliations:** 1 Department of Orthopaedic Surgery, Faculty of Medicine, University of Tsukuba, 1-1-1 Tennodai, Tsukuba 305-8575, Japan; 2 Center for Innovative Medicine and Engineering, University of Tsukuba Hospital, 1-1-1 Tennodai, Tsukuba 305-8575, Japan; 3 Department of Orthopedic Surgery, Tokyo Medical University Ibaraki Medical Center, Ami 300-0395, Japan

**Keywords:** PJK, 3D gait analysis, adult spinal deformity, degenerative lumbar kyphoscoliosis, spinal sagittal alignment, dynamic spinal alignment

## Abstract

Background: Severe spinal deformity is a risk factor for proximal junctional kyphosis (PJK) in surgery for adult spinal deformity (ASD). However, standing X-ray imaging in patients with dynamic spinal imbalance can underestimate the risk of PJK because of compensation mechanisms. This study aimed to investigate whether preoperative dynamic spinal alignment can be a predictive factor for PJK. Methods: We retrospectively included 27 ASD patients undergoing three-dimensional (3D) gait analysis before surgery. Dynamic spinal parameters were obtained using a Nexus motion capture system (Vicon, Oxford, UK). The patients were instructed to walk as long as possible around an oval walkway. The averaged dynamic parameters in the final lap were compared between patients with PJK (+) and with PJK (−). Results: PJK occurred in seven patients (26%). The dynamic angle between the thoracic spine and pelvis was larger in patients with PJK (+) than in those with PJK (−) (32.3 ± 8.1 vs. 18.7 ± 13.5 °, *p* = 0.020). Multiple logistic regression analysis identified this angle as an independent risk factor for PJK. Conclusions: Preoperative thoracic anterior inclination exacerbated by gait can be one of preoperative independent risk factors for PJK in patients undergoing corrective surgery for ASD.

## 1. Introduction

Corrective surgery for adult spinal deformity (ASD) is a principal means to improve the quality of life by restoring whole spinal alignment [1]. Proximal junctional kyphosis (PJK) is a representative complication of corrective surgery for ASD. The incidence of PJK was previously reported as 20–40% [2,3,4,5,6,7]. It occasionally requires extended instrumentation surgery for instrument failure, sagittal malalignment, or neurological compromise, representing a substantial burden for patients. As risk factors for PJK, patient- and surgically related factors such as older patients, low bone mineral density (BMD), severe spinal malalignment, and longer fused range of spinal correction surgery were previously reported [7]. A meta-analysis investigated other risk factors, and over 20 factors were suggested, but they are difficult to apply to clinical practice [8] because severe deformity itself is a characteristic of ASD. Thus, the prevention of PJK remains to be established despite numerous efforts to overcome this complication [9,10,11,12].

Although worsening spinal alignment during gait was reported in patients with ASD [13], it can barely be detected using conventional standing X-ray images. Three-dimensional (3D) gait analysis can provide quantitative assessment of spinopelvic alignment change and the failure of compensatory mechanisms during gait [13,14,15,16]. Among patients with the spinal malalignment that gait exacerbates, static standing X-ray image assessment can result in underestimating severe spinal deformity. Even if these patients are at a high risk of PJK, they cannot be assessed adequately by conventional assessment before surgery. Thus, we hypothesized that PJK would be affected by preoperative dynamic spinopelvic alignment change during gait. In this study, we sought to investigate whether preoperative dynamic spinal alignment aggravated by gait, as determined by 3D gait analysis, can be a risk factor for PJK.

## 2. Materials and Methods

### 2.1. Study Design and Participant Data

We conducted an observational case–control study within a cohort of patients who underwent spine surgery. We retrospectively included patients with ASD who underwent corrective surgery over three spinal levels and gait analysis at our university hospital between December 2015 and March 2020. The radiographic inclusion criteria were as follows: able to stand without any support during X-ray imaging; pelvic incidence minus lumbar lordosis (PI—LL) > 10°; sagittal vertical axis (SVA) > 4 cm; and pelvic tilt (PT) > 20°, as spinal parameters related to sagittal malalignment according to the SRS-Schwab ASD classification [17]. The exclusion criteria were as follows: (1) proximal junctional failure after another spinal surgery; (2) unable to stand alone during X-ray imaging because of pain or weakness in lower extremities; and (3) <1-year follow-up after surgery.

The demographic data included sex, height, body weight, bone mineral density (BMD) by dual-energy X-ray absorptiometry (DEXA), and fused spinal level. ASD is often complicated by vertebral fractures, and the lumbar DEXA is likely to be higher than the actual bone density, which is reported to deviate significantly from the hip DEXA [18]. In addition, lumbar DEXA does not correlate well with the vertebral body failure load [19]. Based on these reports, we used the BMD of the femur because discrepancies due to osteosclerosis after vertebral body fracture were expected. The radiographical assessments are listed in Section 2.2. 

The present study was conducted within an appropriate ethical framework, and in accordance with the Declaration of Helsinki and its contemporary amendments. The study design was approved by the ethics committee of our institute. Written informed consent was obtained from all patients included in this study.

### 2.2. Radiographic Assessment

We assessed the whole spine parameters digitally before surgery as a static evaluation. Spinal parameters included sagittal vertical axis (SVA); thoracic kyphosis (TK, T5-12); lumbar lordosis (LL, L1-S1); pelvic tilt (PT); pelvic incidence (PI); T1 pelvic angle (TPA); coronal Cobb angle of the thoracolumbar and lumbar scoliosis (Cobb); and coronal balance (C7-CSVL, the distance between a C7 plumb line and the center sacral vertical line). PI–LL was calculated from the values obtained. We measured all parameters in the same manner both preoperatively and postoperatively. All patients were asked to stand normally and look straight ahead in the radiographic exam [20]. PJK was defined as a proximal junctional angle (PJA) > 10° soon after operation and >10° progression of PJA [3]. The PJA and postoperative spinal parameters were assessed at 1 year after surgery. Using this definition of PJK, the patients were classified into PJK (+) and PJK (−) groups. There were no patients who required additional surgery before 1 year postoperatively.

### 2.3. Surgical Procedure

All surgeries in this analysis were performed or supervised by the senior authors, who are experienced board-certified spinal surgeons. For the anterior segment from L2 to L5, extreme lateral interbody fusion (XLIF^®^, Nuvasive, SanDiego, CA, USA) with a mini-open technique was conducted to achieve correction. Interbody fusion was also performed at L5–S1 with conventional posterior lumbar interbody fusion. An anterior vertebral body corpectomy with an expandable cage was performed in patients with deformity caused by vertebral collapse. Subsequently, posterior decompression and correction with an open approach and pedicle screw system were performed. Total facetectomy was performed when needed to achieve sufficient correction. Pedicle subtraction osteotomy was not performed in the present series. We did not add hooks, sublaminar taping, cement augmentation, or other preventive surgical techniques to avoid PJK. The chief surgeon decided the upper instrumented vertebra (UIV) and the lower instrumented vertebra (LIV) depending on each case through conference, and the decision was approved as our consensus.

### 2.4. Gait Analysis and Dynamic Spinal Parameter

**Figure 1:**
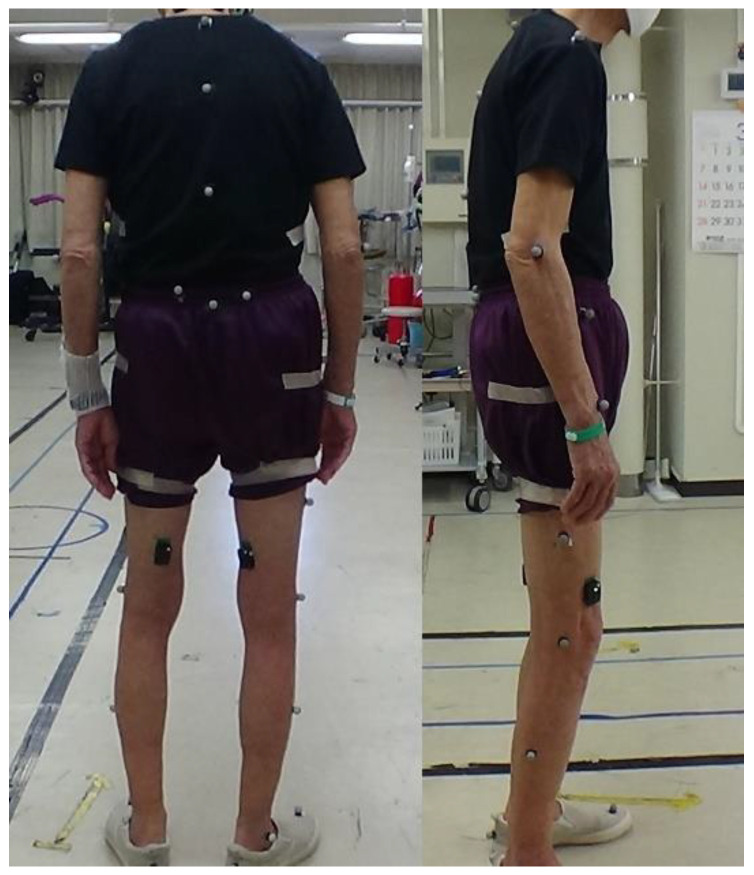
Reflective markers were placed on the spinal spinous and pelvic processes.

The 3D gait analysis was conducted using a Nexus motion capture system (Vicon, Oxford, UK) comprising 16 cameras and 38 reflective markers variously attached on the head, spinal spinous processes, pelvis, and upper and lower limbs of the patients (Figure 1). 

The trials consisted of the patients walking for as long as they could around an oval walkway in a laboratory room. The patients could stop whenever they felt too fatigued to walk any more. The walkway comprised two parallel 10 m straight paths and two semicircular paths of approximately 1 m in radius. Table 1 lists the spinal markers and dynamic spinal parameters obtained from the gait analysis. 

**Figure 2:**
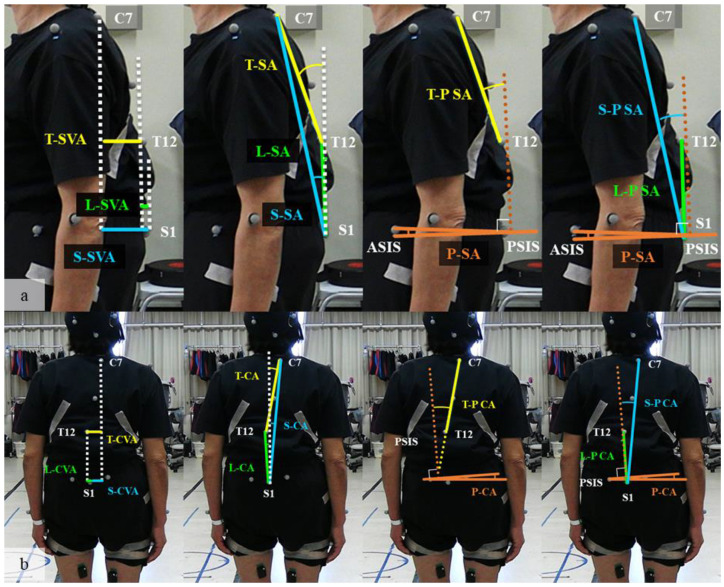
(**a**) Sagittal parameters: the dotted white line indicates a perpendicular line to the floor. The dotted brown line indicates the perpendicular line to the surface created from the two ASIS and two PSIS points (pelvic surface). SVA was defined by the sagittal distance between C7–T12 (T–SVA), T12–S1 (L–SVA), and C7–S1(S–SVA). SA was defined by the sagittal angle of C7–T12 line (T-SA), T12–S1 line (L-SA), and C7–S1 line (S-SA) from the perpendicular line. PSA was defined by the sagittal angle between the floor and the pelvic surface. T-PSA, L-PSA, and S-PSA were defined by the sagittal angle of C7–T12 line (T-P SA), T12–S1 line (L-P SA), and C7–S1 line (S-P SA) from the perpendicular line to the pelvic surface; (**b**) coronal parameters: the dotted white line indicates a perpendicular line to the floor. The dotted brown line indicates the perpendicular line to the surface created from the two ASIS and two PSIS points (pelvic surface). CVA was defined by the coronal distance between C7–T12 (T-CVA), T12–S1 (L-CVA), and C7–S1 (S-CVA). CA was defined by the coronal angle of C7–T12 line (T-CA), T12–S1 line (L-CA), and C7–S1 line (S-CA) from the perpendicular line. PCA was defined by the coronal angle between the floor and the pelvic surface. T-P CA, L-P CA, and S-P CA were defined by the sagittal angle of C7–T12 line (T-P SA), T12–S1 line (L-P SA), and C7–S1 line (S-P SA) from the perpendicular line to the pelvic surface. (T), thoracic; (L), lumbar; (S), whole spinal; SVA, sagittal vertical axis; SA, sagittal angle; P SA, pelvic sagittal angle; ASIS, anterior superior iliac spine; PSIS, posterior superior iliac spine.

Figure 2 summarizes each dynamic spinal parameter. These spinal parameters were recorded continuously during all trials. The mean values of the parameters for each lap were calculated as the parameters for the lap. We sampled the parameters of the final lap, which is considered to exhibit the most exacerbated alignment.

### 2.5. Statistical Analysis

All continuous values are described as mean ± standard deviation (SD). We compared all parameters between the group with PJK (+) and the group with PJK (−) using an unpaired Student *t*-test. A Shapiro–Wilk test was used for each dynamic parameter to evaluate the normal distribution. The post hoc analysis for the Student t-test was performed using G-power software (version 3.1.9.6, Dusseldorf, Germany). Preoperative variables associated with PJK (*p* < 0.10) on univariate analysis were included in a multivariate logistic regression model with forward stepwise algorithms. Variables that did not fit the model significantly were rejected. Odds ratios (ORs) and 95% confidence intervals (CIs) were calculated. A Hosmer–Lemeshow test was used as a statistical test for the goodness of fit of the logistic regression model. All statistical analyses except for the post hoc power analyses were conducted using JMP statistical software for Windows (version 16; SAS, Cary, NC, USA). *p* < 0.05 was considered significant for tests of statistical difference.

## 3. Results

### 3.1. Patient Inclusion and Demographic Data

**Figure 3:**
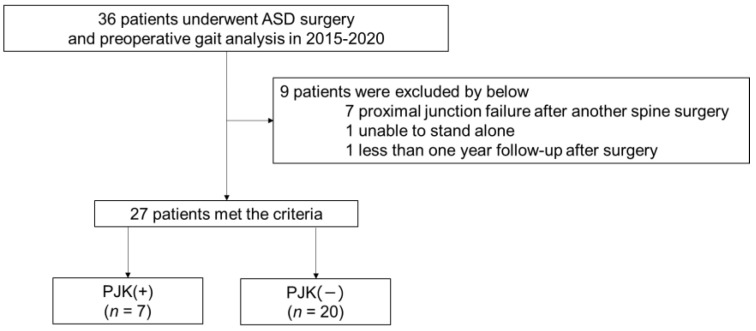
Patient inclusion flow chart. PJK (+) is the patient group with a proximal junctional angle (PJA) >10° soon after surgery and >10° progression of the PJA. PJK (−) is the patient group with a proximal junctional angle (PJA) <10° soon after surgery or <10° progression of the PJA.

First, we included 36 patients with ASD who underwent gait analysis and corrective surgery. Nine patients met the exclusion criteria; therefore, 27 were eventually included in this analysis (Figure 3).

The cohort included seven male and 20 female patients. PJK occurred in seven (26%; one male and six females) of them. There were no significant differences in age, height, body weight, or BMD. In addition, neither sagittal nor radiographic parameters, including C7SVA, TK, LL, and PI–LL, were significantly different preoperatively (Table 2).

Vertebral body corpectomy for osteoporotic vertebral fracture was performed in two patients with PJK (+) and two with PJK (−). In the postoperative assessment, in the standing X-ray images, C7SVA, TK, and LL were significantly greater, and PI−LL was significantly smaller in PJK (+) (TK, 49.3 ± 12.2 vs. 26.3 ± 15.3, *p* = 0.002; LL, 54.0 ± 10.8 vs. 34.5 ± 17.7, *p* = 0.012; PI–LL, −9.0 ± 12.3 vs. 9.4 ± 20.1, *p* = 0.033) (Table 3).

### 3.2. Three-Dimensional Gait Analysis (Dynamic Spinal Parameters)

In the preoperative 3D gait analysis, the thoracic–pelvic spinal angle (T-PSA) in patients with PJK (+) was significantly larger than that in patients with PJK (−) (32.2 ± 8.1 vs. 18.7 ± 13.5°, *p* = 0.020). No significant differences existed in any parameter except for T-PSA (Table 4). 

Other sagittal parameters (T-SVA, L-SVA, S-SVA, T-SA, L-SA, S-SA, L-PSA, and S-PSA) were not significantly different. There was no difference in the dynamic coronal parameters (Table 5). Potential variables (T-PSA and L-PSA levels) were included in the multiple logistic regression model. This model found that T-PSA was an independent preoperative factor significantly associated with PJK (OR, 1.23; 95% CI, 1.031–1.477; *p* = 0.0005). The Hosmer–Lemeshow test result showed that the model was a good fit (*p* = 0.858).

### 3.3. Post Hoc Power Analysis of Student’s T-Test

We performed a post hoc power analysis for unpaired two-group comparisons with significant differences. The effect size was calculated from the mean and standard deviation of the T-PSA of each group and was determined to be 1.48. The effect size and sample size of this analysis indicated that the power (1–β error probability) was 0.90, indicating an adequate sample size.

### 3.4. Representative Case

**Figure 4:**
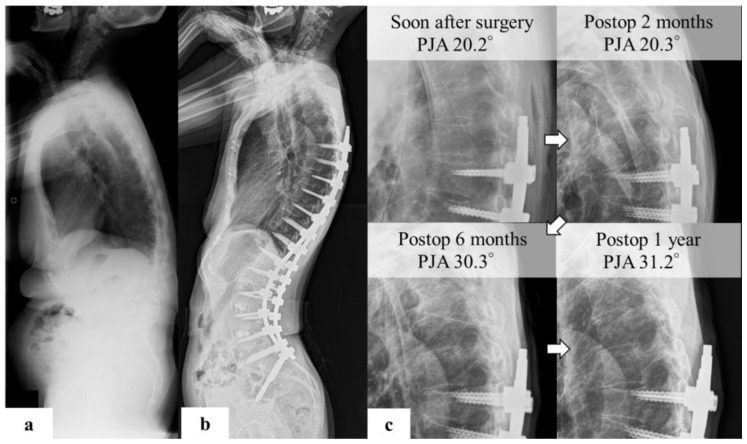
(**a**) Representative case of PJK preoperative standing X-ray images indicated the following spinal parameters: C7SVA, 86 mm; TK, 20°; LL, 20°; PT, 30°; PI, 53°; TPA, 32°; and PI–LL, 23°. (**b**) postoperative images show that the PJA was 20.2. (**c**) PJA developed 6 months postoperatively. The PJA was 31.2° in the first year postoperatively, and the change was 11.2°. This patient complained of implant prominence and pain in the proximal junctional area, but refused reoperation.

A 64-year-old woman with adult spinal deformity and 3D gait analysis before surgery underwent corrective surgery for her main complaint of low back pain. The sagittal parameters on preoperative standing X-ray imaging were as follows: C7SVA, 54.2 mm; TK, 33.1°; LL, 41.7°; PT, 20.7°; PI, 50.2°; TPA, 19.8° (Figure 4a). PJK was detected on postoperative radiographic imaging at 6 months. At the postoperative year 1 follow-up, PJA was 31.2°, and the progression of PJA was 11.2° (Figure 4b,c). 

**Figure 5:**
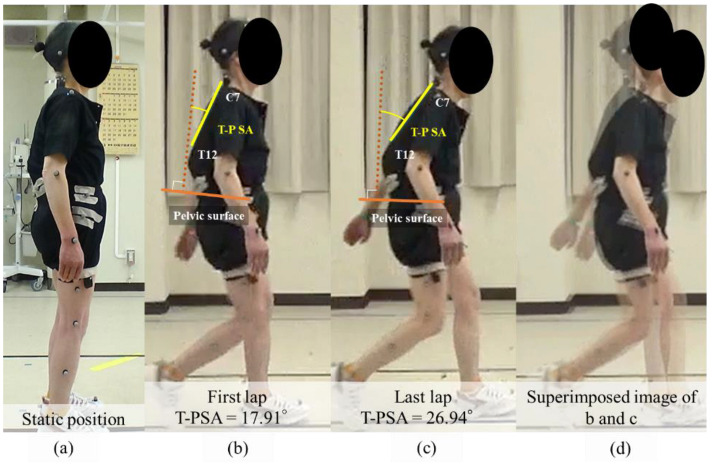
Gait analysis of representative case. This figure shows the posture during gait analysis. Before starting to walk, the patient seemed relatively balanced in an upright standing position before the gait analysis (**a**). When starting the walk, the thoracic region began to lean forward in the first lap (**b**). In the final lap, soon before quitting the trial, the tilt of the thoracic spine leaned further forward (**c**). A superimposed image of the posture during gait showed a change in the thoracic spine tilt between the first (T-P SA 17.91°) and the final lap (T-P SA 26.94°) (**d**).

Figure 5 shows the posture during the gait analysis. Before walking, the patient seemed relatively balanced in a standing upright position before the gait analysis (Figure 5a). The thoracic part began to lean forward in the first lap (Figure 5b). In the final lap, soon before quitting the trial, the tilt of the thoracic spine leaned further forward (Figure 5c). The superimposed image of posture during gait showed a change in thoracic spine tilt between the first (T-P SA 17.9°) and the final lap (T-P SA 27.0°) (Figure 5d). This patient complained of implant prominence and pain in the proximal junctional area, but refused reoperation. 

## 4. Discussion

The present study investigated the association of preoperative dynamic spinal malalignment exacerbated by gait with postoperative PJK incidence. The preoperative T-PSA was larger in patients with PJK (+) than it was in those with PJK (−) with sufficient sample size. The multiple logistic regression analysis revealed that preoperative T-PSA was an independent factor significantly associated with PJK. The preoperative demographic data of both groups indicated no significant difference in age, osteoporosis, or sagittal alignment in the standing X-ray images.

SVA is one of the parameters indicating the severity of whole-spine deformity and is exacerbated while walking [20], but it is directly influenced by compensation from pelvic retroversion and knee flexion. By contrast, T-PSA, which is defined as the angle between the thoracic spine and the pelvis, could subtract the effect of pelvic retroversion and can be interpreted as an independent parameter from compensation by the pelvis and lower extremities. In this study, T-SA, P-SA, and S-SVA, for which pelvis compensation can have an impact, were not different between the two groups, whereas the difference of T-PSA was evident between the PJK (+) and PJK (−) groups. Walking disrupts the compensation because of muscle fatigue, even in the paravertebral muscles [20]. The difference between the two groups may be due to the fact that back muscle failure is more evident in the PJK group than in the no PJK group due to walking fatigue.

A previous 3D gait analysis for PJK showed that excessive pelvic anteversion during short walking periods is an independent risk factor for PJK [21]. It concluded that walking disrupts pelvic compensation, resulting in the more severe whole-spine malalignment than static X-ray assessment. Meanwhile, this study revealed that the patients had an anterior tilt of the thoracic spine despite effective compensation in the lumbar spine and pelvis during gait, suggesting that the sufficient muscular endurance required to properly compensate the thoracic spine, lumbar spine, and pelvis may differ. Previous reports have investigated the relationship between PJK and lower muscularity in representative levels of the paravertebral muscle [21,22], and low muscular endurance in the paravertebral muscle around the thoracic spine can explain this anterior-tilting thoracic spine. The muscularity in each spinal level may vary among patients with ASD, and thus, the lower muscular endurance in the thoracic spine level compared to in the lumbar spine and the lower extremities may cause the compensated, but tilting forward thoracic spine, leading to a large T-PSA, indicating a risk of PJK.

In the postoperative X-ray image assessment, TK and LL were significantly larger and PI-LL was significantly lower in patients with PJK (+). PI–LL < 0 has been reported as a risk factor for PJK [23]. Excessive postoperative LL for PI results in a PI–LL mismatch as well as inadequate correction. Patients with an excessively flexible thoracic spine preoperatively are more likely to have reciprocal change in unfused thoracic spine, leading to the development of PJK. Age-adjusted appropriate alignment is suggested as a goal for ideal alignment to prevent PJK in the elderly [24], while another study emphasized the importance of strict correction to achieve better ODI outcomes, especially in elderly Japanese patients [25]. These studies indicated that we need to consider the optimal alignment on a patient-by-patient basis. Patients with a tendency for the thoracic spine to tilt forward during walking may have an over-reciprocal change after corrective surgery for ASD, but to our knowledge, no previous study has investigated the relationship between spinal alignment during gait and spinal change after surgery. Therefore, adequate alignment in patients with a large T-PSA after walking should be investigated in future study.

Some limitations should be addressed. First, we did not investigate the patient-reported outcome of quality of life (QoL). PJK was reported as having no effect on a patient’s QoL, but subsequent studies have gradually clarified its relationship with QoL. We need to investigate further the relationship between clinical outcome and this gait analysis. Second is the possibility of selection bias. This study included patients with corrective surgery and preoperative gait analysis. It is ideal that all patients underwent gait analysis before corrective surgery, but the indication of gait analysis was determined by the patient and examination room schedule before surgery. Because of this, this patient group had a limited sample size. Power analysis indicated that the sample size was sufficient, but the possibility of a type II error cannot be ruled out. In future study, we need to collect a larger sample size. Finally, the accuracy of the reflective marker on the skin should be considered. To reduce the measurement error, we instructed patients to wear prepared tight clothes and put the markers on them. Soft tissue thickness is difficult to overcome, especially on the lumbar spine [26]. The 3D gait analysis system in the present study included the influence of soft tissue on the accuracy of the marker placement, but the influence of the lumbar lordosis was minimized in the analyzed population because most patients had reduced lumbar lordosis due to ASD.

## 5. Conclusions

The present study indicated that thoracic kyphosis exacerbated by gait, as determined by 3D gait analysis, can be a preoperative independent risk factor for PJK in patients with ASD undergoing corrective surgery. By contrast, lumbar spinal change was not significantly different between patients with PJK (+) or PJK (−). To assess risk, dynamic thoracic anterior tilt should be assessed carefully, rather than lumbar spine alignment alone.

## Figures and Tables

**Table 1:** Dynamic spinal alignment parameters.

Parameter	Definition	Marker		Unit
T-SVA	thoracic sagittal distance between the reflective markers	C7	T12	mm
T-CVA	thoracic coronal distance between the reflective markers	C7	T12	mm
L-SVA	lumbar sagittal distance between the reflective markers	T12	S1	mm
L-CVA	lumbar coronal distance between the reflective markers	T12	S1	mm
S-SVA	whole spinal sagittal distance between the reflective markers	C7	S1	mm
S-CVA	whole spinal coronal distance between the reflective markers	C7	S1	mm
T-SA	thoracic sagittal angle between the vertical axis and the line connecting the spinal markers	C7	T12	°
T-CA	thoracic coronal angle between the vertical axis and the line connecting the spinal markers	C7	T12	°
L-SA	lumbar sagittal angle between the vertical axis and the line connecting the spinal markers	T12	S1	°
L-CA	lumbar coronal angle between the vertical axis and the line connecting the spinal markers	T12	S1	°
S-SA	whole spinal sagittal angle between the vertical axis and the line connecting the spinal markers	C7	S1	°
S-CA	whole spinal coronal angle between the vertical axis and the line connecting the spinal markers	C7	S1	°
P-SA	sagittal angle between the horizontal axis and the line connecting the reflective markers on the ASIS and PSIS	ASIS	PSIS	°
P-CA	coronal angle between the horizontal axis and the line connecting the reflective markers on the ASIS and PSIS	ASIS	PSIS	°
T-P SA	thoracic sagittal angle between the line connecting the spinal markers and the line connecting the reflective markers on the ASIS and PSIS	C7	T12	°
T-P CA	thoracic coronal angle between the line connecting the spinal markers and the line connecting the reflective markers on the ASIS and PSIS	C7	T12	°
L-P SA	lumbar sagittal angle between the line connecting the spinal markers and the line connecting the reflective markers on the ASIS and PSIS	T12	S1	°
L-P CA	lumbar coronal angle between the line connecting the spinal markers and the line connecting the reflective markers on the ASIS and PSIS	T12	S1	°
S-P SA	whole spinal sagittal angle between the line connecting the spinal markers and the line connecting the reflective markers on the ASIS and PSIS	C7	S1	°
S-P CA	whole spinal coronal angle between the line connecting the spinal markers and the line connecting the reflective markers on the ASIS and PSIS	C7	S1	°

SVA, sagittal vertical axis; CVA, coronal vertical axis; SA, sagittal angle; CA, coronal angle; P SA, pelvic sagittal angle; P CA, pelvic coronal angle; ASIS, anterior superior iliac spine; PSIS, posterior superior iliac spine.

**Table 2:** Patient demographics.

	Parameter	PJK (+)		PJK (−)		*p*
*n*		7		20		
Sex (male, female)		1	6	4	16	
Age (years)		67.5	±6.24	68.6	±10.2	0.969
Height (cm)		146.7	±7.8	151.1	±35.3	0.340
Body weight (kg)		52.9	±12.3	49.6	±14.1	0.668
BMD (g/cm^2^)		0.63	±0.1	0.60	±0.2	0.669
YAM (%)		77.3	±8.6	73.6	±25.9	0.635
Preop	C7SVA (mm)	115.9	±51.9	112.7	±58.1	0.900
	TK °	20.5	±11.8	16.3	±15.6	0.524
	LL °	6.3	±16.8	11.7	±20.3	0.703
	PT °	36.7	±9.4	31.6	±14.8	0.436
	PI °	52.3	±12.8	49.6	±15.4	0.501
	TPA °	39.7	±11.4	35.5	±16.7	0.625
	PI–LL °	46.0	±19.5	37.8	±23.2	0.463
	C7CSVL (mm)	3.6	±31.3	4.6	±28.9	0.742
	Coronal Cobb angle°	37.4	±21.7	24.1	±15.5	0.102

Mean ± standard deviation; *n*, number of patients; BMD, bone mineral density; YAM, young adult mean; C7SVA, C7 plumb line to sagittal vertical axis distance; TK, thoracic kyphosis; LL, lumbar lordosis; PT, pelvic tilt; PI, pelvic incidence; TPA, T1 pelvic angle; PI–LL, PI minus LL, C7CSVL, C7 plumb line to central sacral vertical line distance; PreOp, preoperative parameter.

**Table 3:** Postoperative spinal parameters.

Parameter	PJK (+)		PJK (−)		*p*
Fused levels	10.4	±2.0	7.7	±3.7	0.069
C7CSVL (mm)	15.3	±22.9	6.5	±15.2	0.258
Coronal Cobb angle °	10.8	±15.5	10.4	±13.3	0.638
C7SVA (mm)	−8.3	±35.8	40.2	±48.2	0.023 *
TK °	49.3	±12.2	26.4	±15.3	0.002 *
LL °	54.0	±10.8	34.9	±18.3	0.015 *
PT °	19.8	±10.8	19.9	±12.9	0.978
PI °	45.1	±6.6	43.7	±11.2	0.769
TPA °	12.5	±10.8	17.6	±12.6	0.357
PI–LL °	−9.0	±12.2	9.4	±20.1	0.033 *

* *p* < 0.05; CSVL, central sacral vertical line distance; C7S VA, C7 plumb line to sagittal vertical axis distance; TK, thoracic kyphosis; LL, lumbar lordosis; PT, pelvic tilt; PI, pelvic incidence; TPA, T1 pelvic angle; PI–LL, PI minus LL.

**Table 4:** Dynamic sagittal parameters between each group.

Parameter	PJK (+)		PJK (−)		*p*
T-SVA (mm)	158.4	±36.8	118.2	±46.6	0.050
L-SVA (mm)	15.0	±21.3	27.4	±31.8	0.351
S-SVA (mm)	194.8	±56.2	165.1	±74.5	0.347
T-SA °	33.5	±9.2	25.1	±12.1	0.107
L-SA °	4.9	±8.1	10.0	±11.8	0.300
S-SA °	25.0	±7.2	20.4	±9.9	0.278
P-SA °	92.4	±4.4	90.2	±22.3	0.802
T-P SA °	32.3	±8.1	18.7	±13.5	0.020 *
L-P SA °	-1.9	±14.1	5.1	±11.2	0.193
S-P SA °	22.5	±7.2	14.5	±12.1	0.116

* *p* < 0.05. T, thoracic; L, lumbar; S, whole spinal; SVA, sagittal vertical axis; SA, sagittal angle; P-SA, pelvic sagittal angle; -P SA, sagittal angle of each spinal segment to the pelvic surface.

**Table 5:** Dynamic coronal parameters between each group.

Parameter	PJK (+)		PJK (−)		*p*
T-CVA (mm)	10.8	±31.7	8.2	±19.9	0.802
L-CVA (mm)	1.4	±13.8	−3.0	±19.5	0.589
S-CVA (mm)	11.2	±31.4	7.7	±34.6	0.819
T-CA °	3.4	±8.4	2.2	±5.1	0.655
L-CA °	0.6	±5.5	0.4	±9.5	0.960
S-CA °	1.7	±4.6	1.4	±5	0.909
P-CA °	88.5	±7.5	86.1	±20.3	0.764
T-P CA °	5.1	±12.2	−0.2	±11.1	0.305
L-P CA °	−4.2	±11.8	−0.4	±10.7	0.435
S-P CA °	3.1	±9.2	−0.7	±8.2	0.326

T, thoracic; L, lumbar; S, whole spinal; CVA, coronal vertical axis; CA, coronal angle; P-CA, pelvic coronal angle; -P-CA, coronal angle of each spinal segment to the pelvic surface.

## Data Availability

The data presented in this study are available on request from the corresponding author. The data are not publicly available due to patients’ privacy.
